# The New Face of the Old Molecules: Crustin Pm4 and Transglutaminase Type I Serving as RNPs Down-Regulate Astakine-Mediated Hematopoiesis

**DOI:** 10.1371/journal.pone.0072793

**Published:** 2013-08-27

**Authors:** Yun-Tsan Chang, Cheng-Yung Lin, Che-Yiang Tsai, Vinu S. Siva, Chia-Ying Chu, Huai-Jen Tsai, Yen-Ling Song

**Affiliations:** 1 Institute of Zoology, National Taiwan University, Taipei, Taiwan, ROC; 2 Institute of Molecular and Cellular Biology, National Taiwan University, Taipei, Taiwan, ROC; 3 Department of Life Science, National Taiwan University, Taipei, Taiwan, ROC; Temple University, United States of America

## Abstract

Astakine is an important cytokine that is involved in crustacean hematopoiesis. Interestingly, the protein levels of astakine increased dramatically in plasma of LPS-injected shrimp while mRNA levels remained unchanged. Here, we investigated the involvement of astakine 3′-untranslated region (UTR) in its protein expression. The 3′-UTR of astakine down-regulated the expression of reporter protein but the mRNA stability of reporter gene was unaffected. We identified the functional regulatory elements of astakine 3′-UTR, where 3′-UTR_242–483_ acted as repressor. The electrophoresis mobility shift assay (EMSA), RNA pull-down assay and LC/MS/MS were performed to identify the protein association. We noted that crustin Pm4 and shrimp transglutaminase I (STG I) were associated to astakine 3′-UTR_242–483_, while two other proteins have yet to be revealed. Depletion of hemocytic crustin Pm4 and STG I significantly increased the protein level of astakine while astakine mRNA level remained unaffected. Lipopolysaccharide (LPS) stimulated the secretion of crustin Pm4 and STG I from hemocytes to plasma and increased the astakine level to stimulate the hemocytes proliferation. Altogether, we identified the shrimp crustin Pm4 and STG I as novel RNA binding proteins that play an important role in down-regulating astakine expression at post-transcriptional level and are crucial for the maintenance of hematopoiesis.

## Introduction

Crustaceans have open circulatory system in which maintenance of homeostasis and innate immune response are closely related, and where hemocytes play important roles against pathogens [1], [2]. During infection or massive hemocytes loss, hematopoietic tissues produce hemocytes to maintain homeostasis. Some immunostimulants, such as LPS and laminarin, cause massive depletion of hemocyte. After LPS injection, the circulating hemocyte percentage is considerably decreased to 40% within 3 hours but is restored to 100% in 3–24 hours [3]. Meanwhile, the cells significantly proliferate in hematopoietic tissue of tiger shrimp *Penaeus monodon* after LPS injection [4]. These similar observations have been observed using laminarin stimulation [5]. Thus, it is considered that when shrimp is infected by a pathogen, the involvement of hematopoiesis and hemocyte regulation is crucial response to the pathogen and maintenance of homeostasis.

Astakine is an important cytokine involved in the hematopoiesis of crustaceans. There are two astakine molecules, astakine 1 and 2, cloned from crayfish with a sequence difference of 13 extra amino acids insert in astakine 2. As for tiger shrimp, one astakine molecule but two transcripts with various lengths of 3′-UTR have been cloned and reported [6], [7]. Shrimp astakine is more similar to crayfish astakine 2 in amino acid sequence (53% identity), but less similar to crayfish astakine 1 (38% identity). However, shrimp astakine shows functional analogy with crayfish astakine 1 in stimulating hemocyte proliferation in the hematopoietic tissue [6], [7]. As for crayfish astakine 2, it fails to stimulate hemocyte proliferation, instead, it stimulates crayfish hemocyte to further differentiate and mature into granulocytes [8]. Serving as hematopoietic growth factors, astakine 1 may be involved in the early developmental stage and astakine 2 in the late stage in crayfish. In a recent study, the expression of crayfish astakine 2 is up-regulated by melatonin in brain during the dark period of the circadian rhythm [9]. However, the intracellular regulatory mechanism of astakine is still unknown.

In shrimp, the long form astakine 3′-UTR is 2.64 folds longer than its ORF length. After LPS injection, the astakine protein is increased in plasma of crayfish [7], while the mRNA level of astakine remains unchanged in hemocytes of crayfish and tiger shrimp [6], [7]. Hence, it is believed that astakine translational regulation plays a vital role in its gene regulation. Recent studies on the molecular mechanisms of inflammatory responses and hematological disorders in human indicate clearly that the regulation of mRNA translation at the level of translation initiation, mRNA stability, and protein isoform synthesis are involved in the tight regulation of hematopoietic gene expression [10]. Compared to the transcriptional regulation, the post-transcriptional control of existing mRNAs allows for more rapid changes in protein levels during nutrient deprivation and stress, development and differentiation, nervous system function, aging, and diseases [11]–[15]. Regulation through the 3′-UTR of mRNA is one of the critical mechanisms for post-transcriptional control. Moreover, the average length of 3′-UTR has increased during evolution suggesting that its utilization may contribute to organism complexity [16], [17]. In invertebrates, the mean length of functional 3′-UTR is around 300 bp and the extended 3′-UTR length provides potential for transcript-specific regulation [17].

It has been shown that various RNA-binding proteins that interact with 3′-UTR to form ribonucleoprotein (RNP) complexes perform a key role as translational regulator [18]. Besides, diverse RNA-binding protein and association of RNPs to specific recognition elements of mRNAs are part of a pervasive mechanism for multi-dimensional regulation of their post-transcriptional fate [19]. Strikingly, not only the ‘classical’ conventional RNPs but many enzymes with well-established cellular functions can act as nonconventional RNPs, and participate potentially in regulating RNA stability and gene expression [20], [21]. Still there are many conventional and nonconventional RNPs yet to be revealed, and their role in controlling gene expression is important in many aspects, such as invertebrate hematopoiesis. In the present study, for the first time, we identified two nonconventional proteins associated with the regulatory elements in the 3′UTR of astakine and revealed their specific role in regulating the expression of astakine via its 3′UTR242–483.

## Materials and Methods

### RNA secondary structure prediction

The sequence from shrimp long form astakine 3′-UTR (GenBank accession no. EU980444) was submitted to RNA fold web server [22] (http://rna.tbi.univie.ac.at/cgi-bin/RNAfold.cgi) for secondary structure prediction.

### Cell culture

Sf21cell line from *Spodoptera frugiperda* was cultured at 26°C in TNM-FH insect cell medium (Grace's insect cell culture medium (Invitrogen).

Shrimp hemocytes were cultured using primary culture method described by Li et al. [23]. Shrimp hemocytes were drawn from the abdominal segment with a 1 ml syringe containing 0.5 ml anticoagulation solution (0.1 M sodium citrate, 0.4 M sucrose, 0.01 M Tris-HCl, pH 7.6, 780±15 mOsm/kg). Hemocytes were collected by centrifugation, and were then gently suspended with 1 ml Leibovitz L-15 culture medium (Gibco, USA). Hemocytes were counted and distributed into 24-well culture plates (Corning Life Sciences, USA) with 3×105 cells/well. The total volume in each well was adjusted to 500 µl/well using the culture medium, and the culture plates were then placed in a 26°C incubator for 2 h before the next treatment.

### Construction, transfection and activity assay of luciferase by astakine 3′-UTR

Tiger shrimp astakine 3′-UTR was divided into eight segments of different length and named as 3′-UTR1–965, 3′-UTR1–483, 3′-UTR484–965, 3′-UTR1–241, 3′-UTR242–483, 3′-UTRde242–483 (astakine 3′-UTR lacking 3′-UTR242–483), 3′-UTR242–362 and 3′-UTR363–483. All the eight segments were then separately incorporated into pGL3 firefly luciferase reporter plasmid, which has an OpIE2 promoter (pGL3-OpIE2), and the astakine 3′UTR segments were inserted behind the firefly luciferase reporter gene. The phRG *Renilla* luciferase plasmid with TK promoter (phRG-TK) was served as internal control. The pGL3-OpIE2 vector (800 ng/well) and phRG-TK vector (200 ng/well) were co-transfected into *Sf*21 cells by using Cellfectin™ Transfection Reagent (Invitrogen, CA, USA).

After 48 hrs post transfection, the luciferase assay was performed using Dual-Glo luciferase assay system (Promega) with phRG *Renilla* luciferase gene vector as internal control to normalize the transfection efficiency. The firefly and *Renilla* luciferase activities were measured by the Dual-Glo luciferase assay system according to the manufacturer's instructions and the chemiluminescence was read by a Luoroskan Ascent FL (Labsystems) reader. The co-expressed *Renilla* luminescence was used to normalize the firefly luminescence.

### Competition experiment

The astakine 3′-UTR242–483 was subcloned into pEGFP reporter vector with a CMV promoter, and astakine 3′-UTR242–483 was inserted behind the reporter gene. This competitor plasmid was named as pEGFP-CMV-Ast 3′-UTR242∼483.

The pGL3-OpIE2-Ast 3′-UTR242∼483vector (400 ng/well) was co-transfected with various amounts of pEGFP-CMV-Ast 3′-UTR242∼483competitor vector (0 ng/well, 400 ng/well and 1000 ng/well) into *Sf*21 cells by using Cellfectin™ Transfection Reagent (Invitrogen, CA, USA). The pEGFP reporter vector without astakine 3′-UTR242–483 was used as a concentration control vector and the phRG *Renilla* luciferase reporter vector with TK promoter was used as an internal control vector. The procedures for plasmid transfection and luciferase activity assay were performed as above.

### RNA-electrophoretic mobility shift assay (RNA-EMSA) in shrimp hemocyte protein extraction

The astakine 3′-UTR242–483 was subcloned into pCS2+ vector. The constructed pCS2+-Ast 3′-UTR242–483 plasmids were used as template, and SP6 RNA polymerase (Roche) was used for in vitro transcription to synthesize Ast 3′-UTR242–483 RNA according to the manufacturer's instructions. The synthesized Ast 3′-UTR242–483 RNA was biotinylated at 3′ end using Pierce® RNA 3′ End Biotinylation Kit (Pierce, Rockford, IL, USA) according to the manufacturer's instruction.

Live black tiger shrimps (*P. monodon*) were purchased from local vendors in Taiwan. The shrimps were then acclimated for one week before the experimental use. To extract hemocyte protein, freshly prepared hemocytes were homogenized in lysis buffer (10 mM HEPES, 1.5 mM MgCl2, 10 mM KCl, 0.5 mM DTT, treated with 1× protease inhibitor cocktail (Roche), pH 7.9 and incubated at 4°C for 10 minutes. The cells were centrifuged at 10,000 rpm at 4°C for 15 minutes and the clear supernatant was stored at −80°C.

RNA-EMSA was performed using the LightShift® Chemiluminescent RNA-EMSA kit (Pierce, Rockford, IL, USA) according to the manufacturer's instructions. The reaction mixture (20 µL) containing about 3 µg hemocyte extract was incubated with biotin-labeled Ast 3′-UTR242–483 transcripts in reaction buffer for 30 minutes at room temperature. Then, samples were ran onto a 6% nondenaturing polyacrylamide gel and transferred to nylon membrane in 0.5×TBE buffer. The biotin-labeled Ast 3′-UTR242–483 RNA was detected using streptavidin-horseradish peroxidase conjugate and chemiluminescent substrate. The signal was detected by Luminescent image analyzer (FluorChem M).

### RNA binding protein: biotin pull-down assay

For biotin pull-down assay, the biotin-labeled Ast 3′-UTR242–483 transcripts were incubated with 30 µg of total shrimp hemocyte lysate for 30 min at 25°C and then complexes were isolated with Dynabeads® M-280 Streptavidin (Invitrogen). After washing with lysis buffer, the pull-down RNA-binding proteins were analyzed by SDS-PAGE.

### Identification of proteins interacting with Ast 3′UTR_242–483_

To identify the RNA-binding proteins, in-gel digestion was performed. In brief, the protein band in 12.5% SDS-PAGE was manually excised from the gel and sliced into pieces. The gel pieces were incubated for 1 hour with 50 mM DTE in 25 mM ammonium bicarbonate, pH 8.5, at 37°C, and subsequently alkylated for 1 hour with 100 mM iodoacetamide in 25 mM ammonium bicarbonate at 25°C. The pieces were then washed with 50% acetonitrile in 25 mM ammonium bicarbonate, dehydrated with 100% acetonitrile, dried, and rehydrated for 16 h in 10 µL of 25 mM ammonium bicarbonate containing 0.1 µg trypsin (sequencing grade, Promega, USA) at 37°C. Following tryptic digestion, peptides were extracted with 50% acetonitrile containing 5% trifluoro acetic acid with moderate sonication. The extracted peptides were evaporated under vacuum. Thereafter, the digested peptides were desalted firstly by using C18 Zip-Tip and sent for LC/MS/MS analysis. The search for matched peptides was done by Mascot algorithm (www.matrixscience.com).

### RNAi against *P. monodon* STG1 and Crustin Pm4 in primary hemocyte

Specific siRNA sequences directed against *P. monodon* STG I and crustin Pm4 mRNA (GenBank accession no. AY074924.1 and FJ686015.1) were designed and ordered from Sigma. The antisense strand sequences of both siRNAs are: siSTG1: 5′- CUCCUGUGGCCACGGGACCGG-3′; siCru: 5′-UAAACCGCCUCCUAAGCCG- 3′, 5′-AAACCGCCUCCGUUGACAC-3′ and 5′-AACCGCCUCCGUUGACACC-3′ (Table 1).

**Table 1:** Sequences of oligonucleotides used in this study.

Oligo's name	Forward primer (5′-3′)	Reverse primer (5′-3′)	Usage
siSTG I (STG I siRNA)	CCGGUCCCGUGGCCACAGGAG	CUCCUGUGGCCACGGGACCGG	siRNA
siCru (Crustin Pm4 siRNA)	CGGCUUAGGAGGCGGUUUA	UAAACCGCCUCCUAAGCCG	siRNA
	GUGUCAACGGAGGCGGUUU	AAACCGCCUCCGUUGACAC	siRNA
	GGUGUCAACGGAGGCGGUU	AACCGCCUCCGUUGACACC	siRNA
Mock siRNA	AAACCGGUUAGGCCGCAGCGCUCAC	GUGAGCGCUGCGGCCUAACCGGUUU	siRNA
STG I	AAAGCCGGTCCCGTGGCCA	GTTGATCGTCCTCACCTCGCTG	RT-Q-PCR
STG II	CTTCCGTCTCATGTCCCA AGAAGTAGAT	TTCTCCAACTTCGAGAACGATTTCTCCC	RT-Q-PCR
Crustin Pm4	TAACCTGTTCCCACGACTTCA	CCGTAGAAAGAAGGAGGCTTG	RT-Q-PCR
Astakine	GATGCGCAGACTAGGTGACTGTTCT	ATTCCGTGGTAAGAGTCCGTTAGGA	RT-Q-PCR
Hemocytic actin	GCGACGTGGACATCCGTAA	CGATGCCAGGGTACATGGTAGT	RT-Q-PCR

The siRNAs were transfected into shrimp primary hemocytes culture, respectively, using Cellfectin™ Transfection Reagent (Invitrogen, CA, USA). To deplete the target genes, hemocytes were transfected with 200 µl siRNA transfection mixture containing 4 µl Cellfectin™, 30 pmol siRNA, and 196 µl serum-free L-15 medium. In the untreated (UT) well, transfection mixture was replaced with 200 µl of serum-free L-15 medium. For mock transfections, hemocytes were transfected with a mixture of 4 µl Cellfectin™ and 196 µl serum-free L-15 medium. At 5 hours post transfection, the transfection mixture was removed and 300 µL L-15 medium with 16% FBS was added into the wells and all hemocytes were placed in a 26°C humid incubator before the subsequent treatments.

### Hemocytes cDNA and protein preparation

Hemocytes were harvested at 24 hours post transfection and total RNA was extracted using TRIZOL® reagent (Invitrogen) followed by DNase I (Invitrogen) treatment. The cDNA was synthesized using the SuperScript™ III First-Strand Synthesis System for RT-PCR according to the manufacturer's instruction (Invitrogen).

Hemocyte proteins were extracted from siRNA knock-down hemocyte cultures and mock-transfection hemocyte cultures with TRIZOL® reagent (Invitrogen). Proteins were dissolved in cell lysis buffer (7 M urea and 2 M thiourea) and quantified using the Bradford method.

### Real time PCR

The cDNAs were used as template along with STG I, STG II, crustin Pm4 and astakine gene primers (Table 1) and 2×KAPA™ SYBR® qPCR Master Mix (KAPA Biosystems). Real time PCR was performed in an ABI 7500 Q-PCR system using the standard program. The *P. monodon* β-tubulin gene served as an internal control. The relative expression ratio was represented by the equation: (each gene expression level)/(β-tubulin expression level). The data were analyzed using analysis of variance (ANOVA) and Duncan's multiple range test (Duncan's MRT) to determine differences between groups. The specificity of real-time PCR products was confirmed by melting curve analysis.

### LPS Injection

The shrimps were injected at the second abdominal segment with lipopolysaccharide (LPS, *E. coli* 055:B5; Sigma-Aldrich). Hemolymph from each of the four shrimps was first withdrawn with anticoagulant as untreated sample and then injected with LPS (1 µg/g shrimp) dissolved in MCHBSS (Modified Complete Hank's Balanced Salt Solution) (10 mM CaCl2, 3 mM MgCl2, 5 mM MgSO4, 24 mg mL−1HBSS (Sigma); 780±15 mOsm kg−1). Hemolymph was again withdrawn from each of the four shrimps at 3 hours post-injection using a syringe with anticoagulant.

### Western blotting

The New Zealand white rabbits were given intra-spleen injection with rAst [7], crustin Pm4 peptide (GSGTYGGGGSYGGGGSYGGC) and STG I peptide (VATGGFFKSD), respectively, five times with 2-week intervals and the antiserum was separated from the blood of the rabbit (Genomics, Taiwan).

The hemolymph and the hemocyte or plasma protein were analyzed by electrophoresis in 15% sodium dodecyl sulfate polyacrylamide gel electrophoresis (SDS-PAGE). The gel was transferred to a PVDF membrane for Western blot, where primary antibody such as STG I antibody (1∶1000 dilute), crustin Pm4 antibody (1∶1000 dilute), rAst antibody (1∶10000 dilute) and GAPDH antibody (1∶5000 dilute; GeneTex) were used. Goat anti-rabbit IgG conjugated alkaline phosphatase antibody (1∶1000 dilute; Abcam; USA) was used as secondary antibody. TBS containing 4-Nitro blue tetrazolium chloride and 5-Bromo-4-chloro-3-indolyl-phosphate (NBT/BCIP) stock solution (Roche) were used for the color development in the dark.

The membrane was scanned and quantified by MataMorph® version 7.0 software. The relative expression ratio was defined as the expression level of STG I, crustin Pm4 or astakine to GAPDH. The data were analyzed using analysis of variance (ANOVA) and Duncan's multiple range test (Duncan's MRT) to determine differences between groups.

## Results

### Characterization of the regulatory element in astakine 3′-UTR

**Figure 1:**
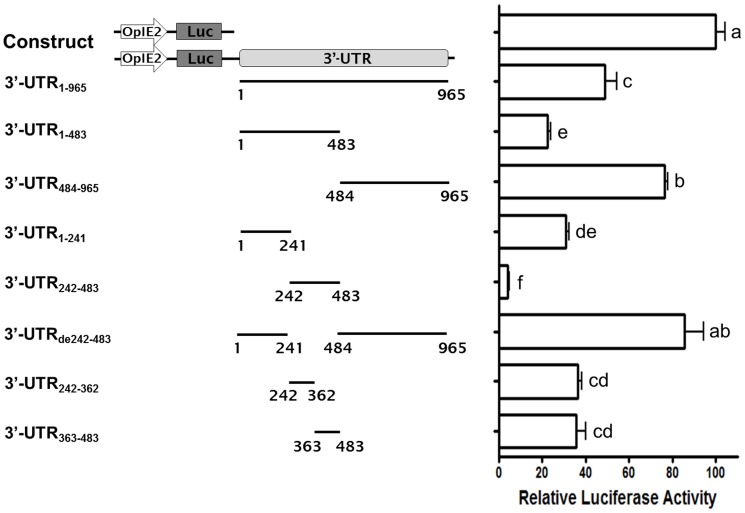
Characterization of regulatory element in astakine 3′-UTR. Luciferase activity assay in *Sf*21 cells transfected with reporter constructs. Eight fragments containing various regions of astakine 3′-UTR were constructed into pGL3-OpIE2, a firefly luciferase reporter vector with the OpIE2 promoter. Relative luciferase activity for each construct was measured and normalized to that of pGL3-OpIE2 empty vector without 3′-UTR (n = 6).

To examine whether shrimp astakine 3′-UTR can regulate upstream gene expression, the full-length astakine 3′-UTR was subcloned into pGL3-OpIE2 vector. We observed that the full length of astakine 3′-UTR down-regulated firefly luciferase activity to about 50% relative to pGL3-OpIE2 vector without whole astakine 3′-UTR (Fig 1, lane 1 and 2). To identify the key regulation segment of astakine 3′-UTR, various regions of astakine 3′-UTR were amplified and subcloned into pGL3-OpIE2 vector for the luciferase reporter assay in *Sf*21 cells. The main regulatory region of astakine 3′-UTR was located in 3′-UTR242–483, which down-regulated the firefly luciferase activity to about 5% relative to pGL3-OpIE2 vector without 3′-UTR (Fig 1, lane 6). To examine the function of the astakine 3′-UTR242–483, we subcloned 3′-UTRde242–483 (astakine 3′-UTR lacking 3′-UTR242–483) into pGL3-OpIE2 vector, and measured the luciferase activity in *Sf*21 cells. Based on the results shown in Fig. 1, the 3′-UTRde242–483 effected the down-regulation of reporter activity and even increased the luciferase activity to higher than that of the full-length astakine 3′-UTR. When we divided the 3′-UTR242–483 into 3′-UTR242–362 and 3′-UTR363–483, and assayed the regulatory function separately, the down-regulation activity was restored to 35%. These data indicate that the key regulatory region of astakine 3′-UTR is located at 3′-UTR242–483. Interestingly, other regions such as 3′-UTR484–965 may act as an enhance element, which can increase the reporter activity to around 30% as compared to full-length astakine 3′-UTR construct.

### mRNA stability assay

**Figure 2:**
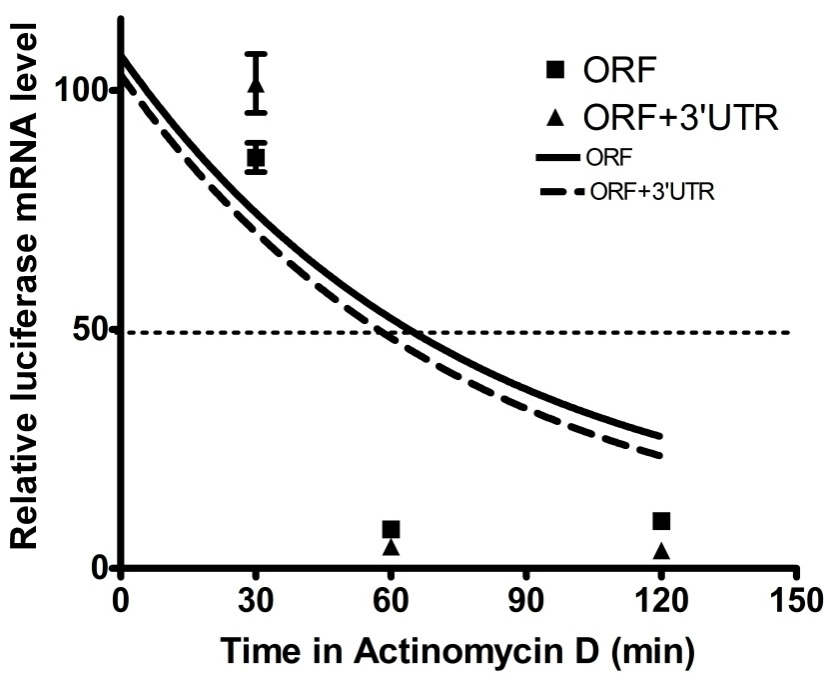
Astakine 3′-UTR effect on mRNA stability. The pGL3-OpIE2 vector with or without full-length astakine 3′-UTR was transfected into *Sf*21 cell and their RNA stabilities were measured. The estimated half-life (t_1/2_) of luciferase RNA with or without astakine 3′-UTR was 60 min and 63 min, respectively.

In order to understand whether astakine 3′-UTR regulated the upstream mRNA stability, pGL3-OpIE2 vectors, with or without full-length astakine 3′-UTR, were transfected into *Sf*21 cell and their RNA stabilities were measured. RNA was extracted at 0, 30, 60 and 120 min after adding actinomycin D and their relative luciferase mRNA levels were measured using RT-qPCR. The two fitting curves almost overlapped, and the estimated half-life (t_1/2_) of RNA for the cells transfected with pGL3-OpIE2 vector containing or not containing full-length astakine 3′-UTR were 60 min and 63 min, respectively (Fig. 2). This result showed that astakine 3′-UTR would not affect the mRNA stability.

### Prediction of astakine 3′-UTR RNA structures

**Figure 3:**
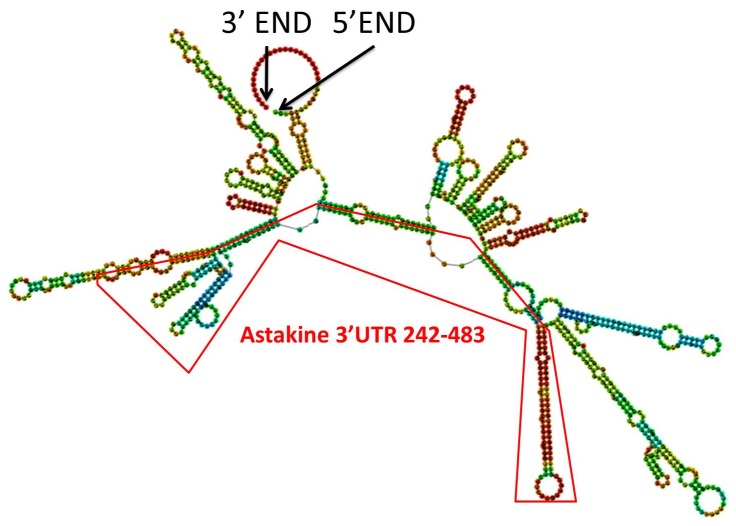
Prediction of astakine 3′-UTR RNA. The secondary structure of full-length astakine 3′-UTR was predicted by RNA fold. In the region of 3′-UTR_242–483_ where the putative regulatory sequence is located, two major secondary loop structures were found.

To investigate the secondary structure of astakine 3′-UTR, the sequence of long form astakine 3′-UTR was submitted for RNA structure prediction. The astakine 3′-UTR sequence is highly structured (Fig. 3). In the region of 3′-UTR242–483 where it exhibits the regulatory activity, we found two secondary loop structures. We hypothesized that the predicted secondary structure could be recognized by specific RNA-binding proteins (RBP) and might be involved in the regulation of astakine protein expression.

### The variation of luciferase activity after co-transfection with competitor plasmid contains astakine 3′-UTR_242–483_

**Figure 4:**
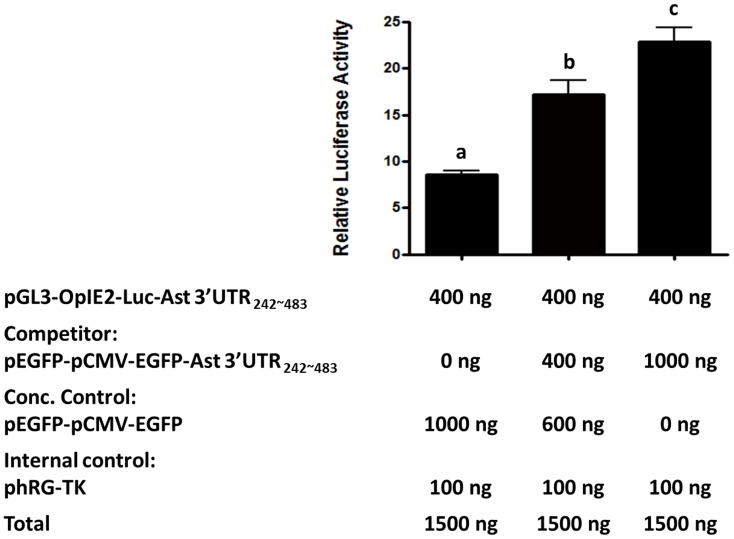
The down-regulation of reporter activity by astakine 3′-UTR_242–483_ in *Sf*21 cells co-transfected with vector expressing astakine 3′-UTR as the competitor. Luciferase activity assay was conducted in *Sf*21 cells which was co-transfected with plasmid pGL3-OpIE2-Ast 3′-UTR_242∼483_ and competitor plasmid pEGFP-CMV-Ast 3′-UTR_242∼483_. Luciferase activity is expressed relative to pGL3-OpIE2 vector without 3′-UTR and data represent mean ±S.D. (n = 6). Different letters represent statistically significant differences as compared to each plasmid-transfected *Sf*21 cell according to the Duncan's multiple range test (*p*<0.05).

To confirm this prediction, we performed competition assay using competitor plasmid pEGFP-CMV-Ast 3′-UTR242∼483. Various ratios of two plasmids, pGL3-OpIE2-Ast 3′-UTR242∼483 and pEGFP-CMV-Ast 3′-UTR242∼483, were co-transfected into *Sf*21 cells then the relative luciferase activity was measured. The results revealed that the luciferase activity for reporter was increased with respect to the increase in competitor plasmid, suggesting the relief of repression through 3′-UTR242–483 (Fig. 4). With the high amount of 3′-UTR242–483 of competitor mRNA, fewer regulatory factors can bind to the 3′-UTR242–483 of firefly luciferase mRNA. Hence, the firefly luciferase activity was restored.

### Multiple protein complexes bind to astakine 3′-UTR_242–483_

**Figure 5:**
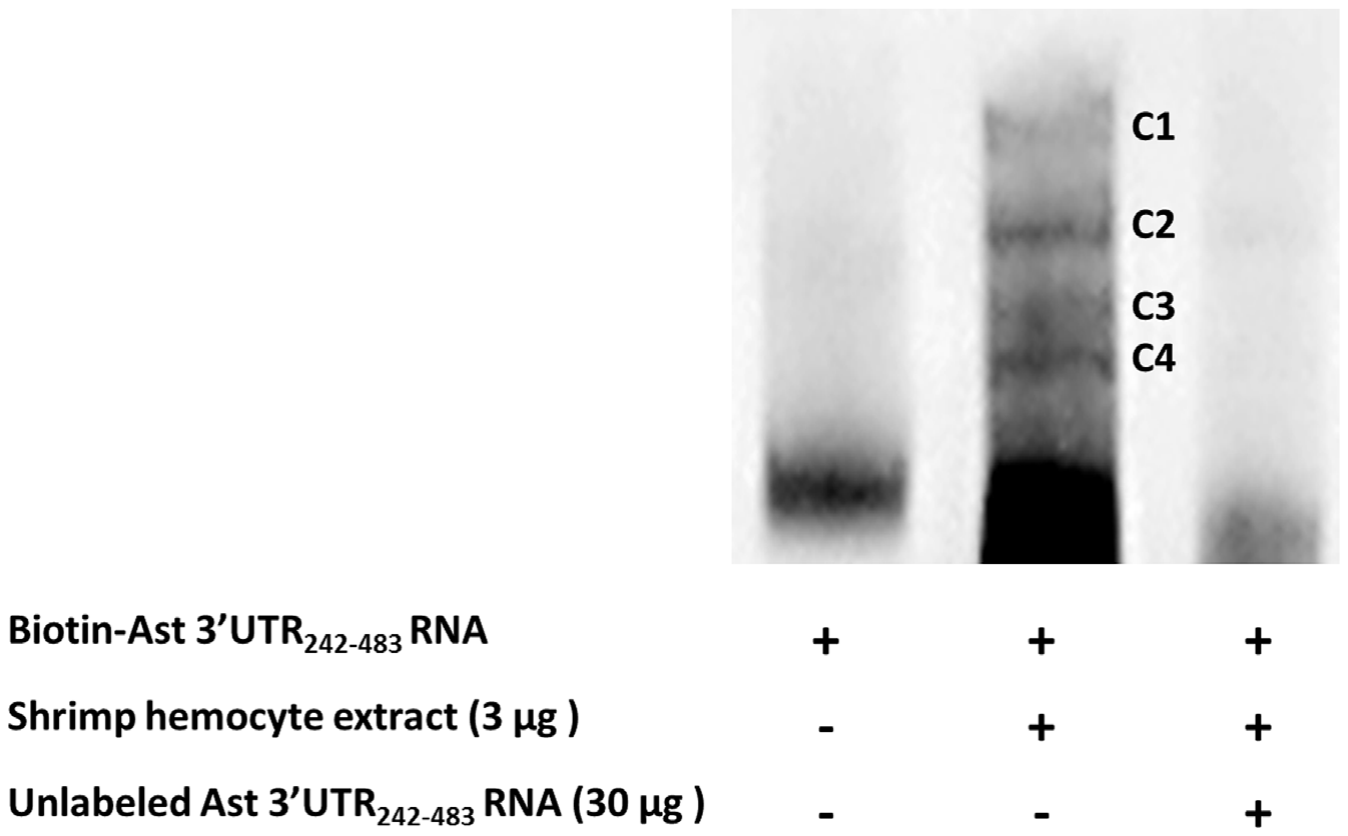
Multiple protein complexes are associated with Ast 3′-UTR_242–483_. RNA EMSA analysis of Ast 3′-UTR_242–483_RNA incubated with shrimp hemocyte extracts shows that four protein complexes are associated with Ast 3′-UTR_242–483_ (C1–C4).

To characterize the RNA binding proteins at astakine 3′-UTR242∼483, we performed RNA-EMSA. The astakine 3′-UTR242–483 transcript was synthesized *in vitro* and incubated with cytoplasmic protein extracts prepared from shrimp hemocyte. Four protein complexes, C1–C4, with various molecular weights were detected by RNA-EMSA (Fig. 5). The specificity of the interaction between astakine 3′-UTR242–483 transcripts and binding protein complexes was confirmed by adding the unlabeled probe as competitor. The amount of binding protein complexes to biotin-labeled astakine 3′-UTR242–483 transcripts was reduced after the addition of the unlabeled astakine 3′-UTR242–483 transcripts.

To identify specific RNA-binding proteins in C1–C4, biotin-labeled Ast 3′-UTR242–483 transcripts were used to pull down the RNA-binding proteins from shrimp hemocytes and were analyzed by LC/MS/MS. The molecular weights of the four detected proteins were determined as 25 kDa, 35 kDa, 70 kDa or 100 kDa. After LC/MS/MS analysis, two proteins were identified as crustin Pm4 (25 kDa) and STG I (100 kDa) and the other two proteins were unidentified (Table 2). Crustin Pm4 and STG I represented as candidates for further investigation.

**Table 2:** Proteins identified by LC/MS/MS.

No.	Gene name	Matched Gene ID	Mass (kDa)	Score
1	Transglutaminase [*Penaeusmonodon*] (STG I)	gi|33694274	84.66	823
2	Unknown protein	-	∼70	-
3	Unknown protein	-	∼35	-
4	Crustin Pm4 antimicrobial peptide [*Penaeusmonodon*]	gi|229459067	24.229	154

### Depletion of STG I and crustin Pm4 does not affect the astakine mRNA

**Figure 6:**
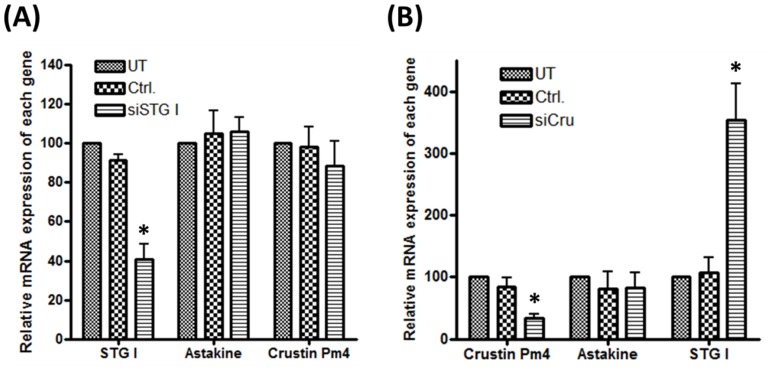
Depletion of STG I and crustin Pm4 does not affect the mRNA level of astakine. (A) Knock-down of hemocytic STG I mRNA after siSTG I transfection. siSTG I significantly decreased STG I mRNA level (n = 8), while no effect was found on the mRNA of astakine. (B) Knock-down of hemocytic crustin Pm4 mRNA expression after siCru transfection. siCru significantly decreased the level of crustin Pm4 mRNA but did not affect astakine mRNA (n = 8). Knock-down of hemocytic crustin Pm4 mRNA increased the level of STG I mRNA. Data represent mean ±SD. Different letters represent statistically significant differences, Duncan's multiple range test (*p*<0.05).

*P. monodon* STG I-specific siRNA, named as siSTG I, was employed for knock-down assay. Compared to untreated group (UT) and mock transfected group (Ctrl.), the depletion of STG I significantly decreased STG I mRNA level to 40% but did not affect the astakine (Fig. 6A) and STG II mRNA level.

To deplete the crustin Pm4 in shrimp, siCru was used. Compared to untreated group (UT) and mock transfected group (Ctrl.), siCru significantly decreased crustin Pm4 mRNA expression to 40% and did not affect the astakine mRNA expression. Interestingly, knock-down of hemocytic crustin Pm4 mRNA induced STG I mRNA expression to about 3.5-fold (Fig. 6B).

### Depletion of STG I and crustin Pm4 increased the protein level of astakine

**Figure 7:**
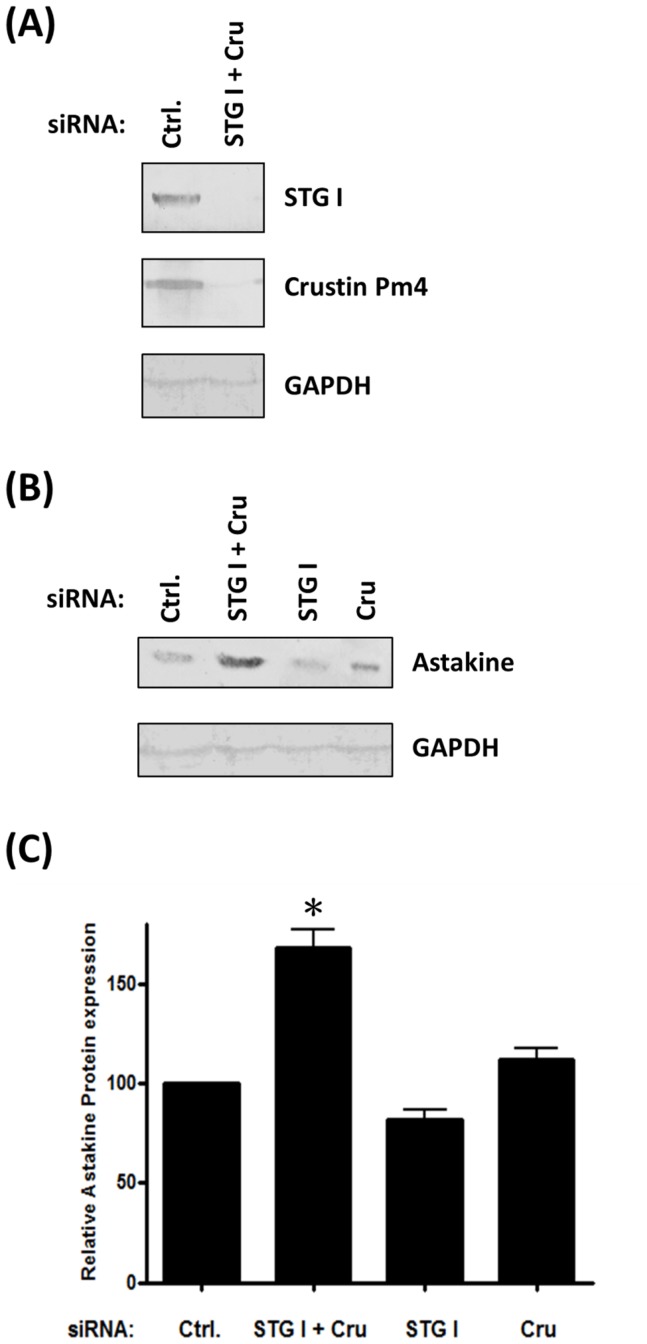
Co-depletion of STG I and crustin Pm4 increases the protein level of astakine. (A) siSTG I and siCru co-transfected hemocytes demonstrate a decrease in protein levels of STG I and crustin Pm4. (B) The proteins from siRNA transfected primary cultured hemocyte and medium were collected and extracted for Western blot of astakine expression. (C) The relative expression of astakine protein was quantified by MataMorph® v7.0 software using GAPDH as internal control (n = 7). Astakine protein expression increased after siSTG I and siCru co-transfection. Data represent mean ±SD. Symbol ‘*’ represent statistically significant difference, Duncan's multiple range test (*p*<0.05).

The knock-down of STG I and crustin Pm4 was assayed at protein level by Western blot using anti-STG I and anti-crustin Pm4 antibody. The examination of STG I and crustin Pm4 protein expression demonstrated a clear reduction in protein after transfection with siSTG I and siCru simultaneously (Fig. 7A). Western blot for astakine showed that the level of astakine protein was increased to 1.6-fold after STG I and crustin Pm4 double knock-down. However, there was no significant change of astakine protein level after STG I or crustin Pm4 knock-down (Fig. 7). These data showed that crustin Pm4 and STG I would act as functional redundancy in astakine expression at the post-transcriptional level.

### LPS stimulation released STG I and crustin Pm4 from hemocyte in vivo

**Figure 8:**
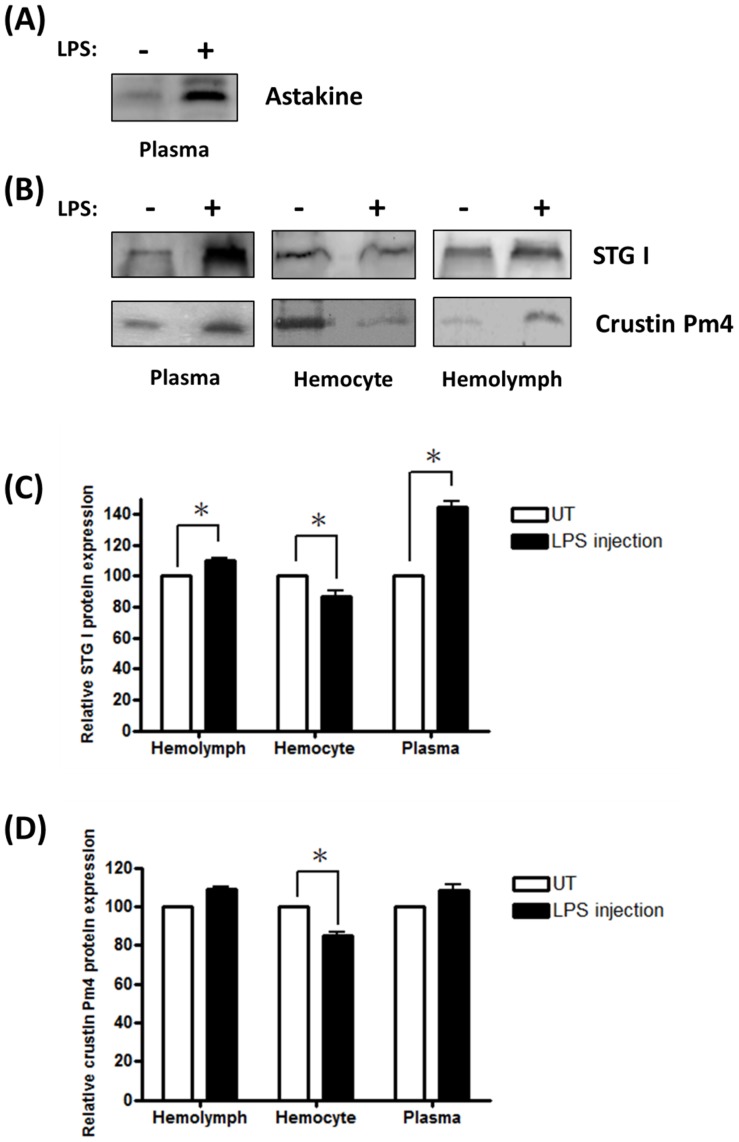
LPS injection induces STG I and crustin Pm4 released from hemocytes *in vivo*. (A) Western blot of astakine in plasma after LPS injection. LPS injection induces increased amount of astakine in the plasma. (B) Western blots of STG I and crustin Pm4 in hemolymph, hemocyte and plasma after LPS injection. The relative expression of STG I (C) and crustin Pm4 (D) protein was quantified by MataMorph® v7.0 software (n = 4). STG I and crustin Pm4 were considerably decreased in hemocyte and STG I protein increased in hemolymph and plasma after LPS injection. 40 µg of proteins from extracted protein were loaded on SDS-PAGE. Data represent mean ±SD. Symbol ‘*’ represent statistically significant difference, Duncan's multiple range test (*p*<0.05).

In order to understand the regulatory mechanism during LPS injection, Western blotting assay was employed to compare the protein level expression of regulatory proteins crustin Pm4 and STG I in hemolymph, hemocyte and plasma of both LPS-injected and untreated shrimp. The result showed that LPS injection increased the amount of astakine in the plasma (Fig. 8A) and both STG I and crustin Pm4 were considerably decreased in hemocyte after LPS injection (Fig. 8). However, STG I protein was increased in the hemolymph and plasma (Fig. 8). The data revealed that two astakine translational repressor proteins were released from hemocytes to plasma after LPS injection.

## Discussion

Astakine, an endocrinal cytokine, is an important humoral factor that regulates crustacean hematopoiesis [6], [24]. Two forms of shrimp astakine transcripts have been reported, with various lengths of 3′-UTR for each form. The additional 671 bp in the 3′-UTR of long astakine transcript suggests that the inserted fragment of 3′UTR may play an important role in regulating the expression of astakine. In this present study, we provided evidence of how astakine is regulated at post-transcriptional level in the immune response via its inserted 3′-UTR.

Because no stable shrimp cell line is available at present, we used the insect *Sf*21 cell line to perform the reporter assay for astakine 3′-UTR in this study. We have established the reporter constructs with OpIE2 promoter that can be expressed in *Sf*21cells [25]. Therefore, the reporter assay in *Sf*21 cell should recapture the 3′UTR activities of shrimp astakine. Our data suggest that the full length 3′-UTR of the long astakine transcript can down-regulate the expression of reporter gene to around 50% at protein level compared to the control. To further characterize the function of astakine 3′UTR, various deletion mutants of astakine 3′UTR were constructed in the reporter system to find the localization of the repressor as well as enhancer of the transcript. Our results revealed that certain regions function as repressors or enhancers, which can fine-tune the expression of astakine. The main negative regulatory element was in 3′-UTR242–483nt, which down-regulated the reporter gene expression to around 92% at protein level. 3′-UTR484–965nt acted as an enhancer to restore the expression level of the reporter gene. This was clearly determined by the specific construct of 3′-UTR484–965nt, and also by the deletion of repressor region 3′-UTR242–483nt. Moreover, the repressor region was further analyzed by reporter assay for the 3′-UTR242–362nt and 3′-UTR363–483nt constructs. Our results showed that the repressor activity was relieved in both constructs compared to 3′-UTR242–483, which may be due to disruption of its secondary structure. Altogether, we identified the main repressor region in astakine 3′-UTR, the 3′-UTR242–483. Surprisingly, this region was localized inside the inserted 671 bp sequence which is only found in the long-form astakine transcripts. Apart from this repressor region, we believe that other regions of 3′UTR are also involved in regulating the expression of astakine through multiple mechanisms. In this study, we are interested in further dissecting the regulatory mechanism of 3′-UTR242–483 because this region down-regulates almost 90% of astakine protein expression.

The translational control mechanisms are modulated via the interaction of RNA binding proteins (RBPs) [17], [26]–[29] or microRNA[30] at 3′-UTR of mRNA. The stability and the rate of the mRNA translation are strictly regulated by some specific RBPs. For example, the half-life and translational rate of the transcripts which contain AU-rich element at the 3′-UTR is controlled by specialized RBPs [27], [28], [31] or the mRNA decay is promoted by ARE-RBPs such as AU-binding factor 1 [31]–[33]. Some RBPs like Hu proteins and NF90 are involved in increasing the mRNA stability and also modulating the translation [34], [35]. However, our result showed the half-life or stability of RNA was not affected by astakine 3′-UTR (Fig. 2). Meanwhile, we found neither AU-rich element nor microRNA binding sites within astakine 3′-UTR from microRNA database. From the 3′-UTR RNA structure prediction, we found several secondary loop structures that could be recognized by RNA binding proteins. Reportedly, some RBPs like TIA-1 and TIAR or FUBP3 have been used to suppress or enhance the translation, respectively [36]–[38]. Hence, a competition assay was conducted to confirm the involvement of astakine 3′-UTR-RBP. Our results suggested that some proteins might have been involved in regulating the expression of astakine, because the competition between two constructs, one of which was 3′-UTR242–483, significantly affected the repression of luciferase gene expression.

To further corroborate this evidence, RNA-EMSA assay was performed to verify the RNA-protein interaction. Results showed four protein complexes with different molecular sizes were associated with 3′-UTR242–483. To disclose the protein information, RNA-pull down analysis was employed, and it revealed the participation of four proteins, which were specifically bound to 3′-UTR242–483. Two of these four proteins, the shrimp transglutaminase I (STG I) and crustin Pm4, had been identified in the shrimp. However, two other proteins were not recognized via LC/MS/MS analysis because the shrimp genome database has not been completed yet. Therefore, the N-terminal sequencing and cloning are necessary to get further information regarding these unknown proteins.

Crustins are among the most important antimicrobial peptides (AMPs) found in decapod crustaceans. Crustin Pm4 is classified as a member of crustin family, but the molecular weight is larger than other crustin Pm isoforms, and its antimicrobial activity is still unknown. Although the antimicrobial peptide or other peptides can nonspecifically bind to DNA or RNA [39], we found only one antimicrobial peptide, crustin Pm4, from our RNA pull down assay. Recent studies showed that antimicrobial peptides have multiple functions and participate not only in antimicrobial function but also in other physiological functions. For example, shrimp penaeidin reportedly behaves as a cytokine for attracting penaeidin-positive granulocytes to the wound tissue, thus, it functions as an autocrine to repair the damaged tissue [23], [40]. LL37, an AMP, allegedly interacts with dsRNA to enhance the TLR3 response against poly(I∶C) and viral dsRNAs [39]. As far as STGs are concerned, TGs are a family known for their roles in blood coagulation. There are two types of TGs, STG I and STG II, have been cloned and identified from shrimp *P. monodon* and STG II plays the roles in blood coagulation. However, STG I does not exhibit coagulation activity in our previous studies [41], [42]. STG I protein has been found to be abundant in hemocytes and high levels of STG I mRNA expression have been detected in hematopoietic tissue based on our previous study [41]. Recently, crayfish *P. leniusculus* TG was preventing the differentiation and migration of hematopoietic stem cells where astakine decreased the TG activity [43]. The amino acid identity of *P. leniusculus* TG closed to *P. monodon* STG II, but the function of STG I was not fully revealed yet. Hence, it is not surprising that STG I, as a non-conventional RNP, has one of the notable functions to control the intricate mechanisms. In this study, we provided evidence that crustin Pm4 and STG I are two known proteins with novel function as nonconventional RNPs participating in astakine repression by interacting with its 3′UTR242–483.

Knock-down of crustin Pm4 and STG I was associated with an increase in astakine protein expression, but no effect on astakine mRNA expression. The result supported our hypothesis that astakine regulation was in translational level, not in transcriptional level. At the same time, there were no differences found in the astakine protein expression with respect to knock-down of crustin Pm4 and STG I individually. Therefore, both crustin Pm4 and STG I were important for the repression of astakine. With the crustin Pm4 knock-down the mRNA expression of STG I was significantly increased, but the upregulation of crustin Pm4 mRNA expression did not happen with the STG I knock-down. It was speculated that when crustin Pm4 was down-regulated, STG I would be highly expressed in order to repress astakine protein expression. Hence, crustin Pm4 was a major protein that collaborated with STG I as RNP rather than an enzyme that participated in astakine repression because we could not find the TG catalytic sites on crustin Pm4, and STG I enzyme activity was very low [41]. In addition to crustin Pm4 and STG I, two unknown RNA binding proteins were not identified yet. The function and relationship of these two proteins with crustin Pm4 and STG I in astakine regulation system should be further investigated. Expression of shrimp astakine is shown to be down-regulated by binding of hemocytic proteins, crustin Pm4 and STG I to astakine 3′-UTR at post-transcriptional level. Interestingly, the expression of crayfish astakine 2 is up-regulated by melatonin, which affects the core clock of crayfish brain, during the dark period of circadian rhythm [9]. Whether expression of the intracellular crustin Pm4 and STGI proteins will be affected during the dark period of circadian rhythm is worthy to further study.

LPS causes the hematopoiesis phenomenon in shrimps such as cell proliferation in hematopoietic tissue [4], down regulation of total hemocyte count (THC), and normalization of hemocyte count after few hours [3]. In addition, LPS injection can also induce the increased amount of astakine protein in plasma [7]. In this study, we confirmed that the increased amount of astakine protein in plasma after LPS injection was because of the regulatory proteins. Once the regulatory proteins, crustin Pm4 and STG I, are secreted from hemocyte to plasma, the translation of astakine mRNA is not repressed, and the increasingly secreted astakine influences the hematopoietic tissue for the production of hemocytes to maintain homeostasis. This mechanism and physiological function of crustin Pm4 and STG I secretions from hemocytes to plasma after LPS injection also need further investigation.

In conclusion, we found that crustin Pm4 and STG I interacted with astakine transcript at 3′-UTR242∼483 and functioned as a nonconventional RNP to down-regulate the astakine protein expression. Furthermore, the depletion of crustin Pm4 and STG I resulted in increasing the astakine protein expression but did not affect its mRNA expression. These results revealed that crustin Pm4 and STG I regulate astakine protein expression through a mechanism at post-transcriptional level which provides new aspect for gene regulation in crustacean immune response.
