# Correction: The New Face of the Old Molecules: Crustin Pm4 and Transglutaminase Type I Serving as RNPs Down-Regulate Astakine-Mediated Hematopoiesis

**DOI:** 10.1371/journal.pone.0182405

**Published:** 2017-07-27

**Authors:** Yun-Tsan Chang, Cheng-Yung Lin, Che-Yiang Tsai, Vinu S. Siva, Chia-Ying Chu, Huai-Jen Tsai, Yen-Ling Song

The authors would like to correct Fig 7, as errors were introduced in the preparation of this figure for publication. The GAPDH band in the Ctrl. lane of Fig 7A is the same as the GAPDH band in the Cru lane of Fig 7B. The authors apologize for the mismatch of astakine expression and its GAPDH expression and note that the mismatch will not influence the conclusion of the article since they loaded the same amount of proteins for every group and confirmed again with GAPDH expression by MataMorph(r) version 7.0 software. The GAPDH expression for every group was almost the same.

**Fig 7:**
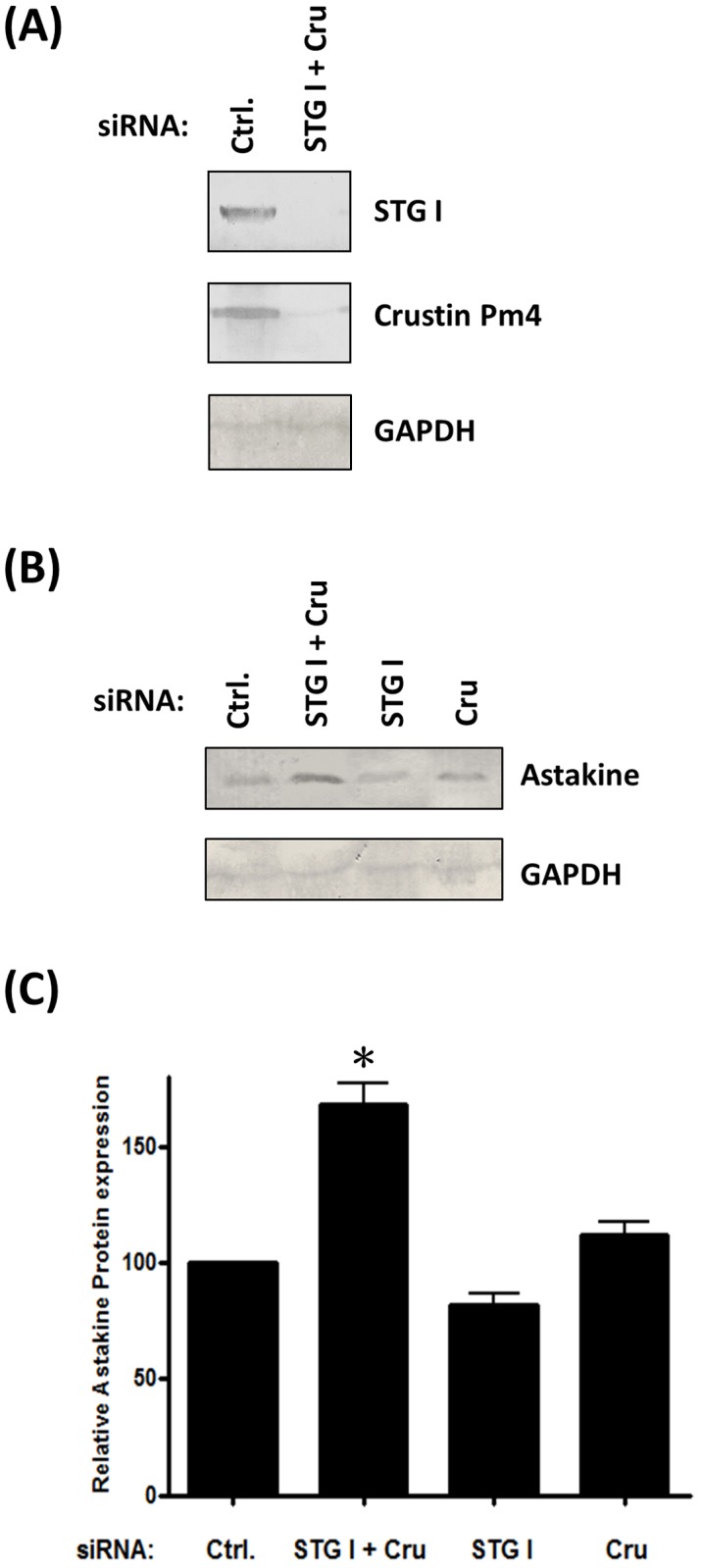
Co-depletion of STG I and crustin Pm4 increases the protein level of astakine. (A) siSTG I and siCru co-transfected hemocytes demonstrate a decrease in protein levels of STG I and crustin Pm4. (B) The proteins from siRNA transfected primary cultured hemocyte and medium were collected and extracted for Western blot of astakine expression. (C) The relative expression of astakine protein was quantified by MataMorph^®^ v7.0 software using GAPDH as internal control (n = 7). Astakine protein expression increased after siSTG I and siCru co-transfection. Data represent mean ±SD. Symbol ‘*’ represent statistically significant difference, Duncan's multiple range test (p<0.05).

The authors transferred the proteins to PVDF membranes and cut the membranes into two or three small membranes depending on which protein they wanted to detect. The authors have provided a Supporting Information file with the entire, full scan and uncut small membranes and all the membranes with markers which can indicate the size of each protein. Please see the corrected Fig 7 here.

## Supporting information

S1 File Uncropped blots. (PDF)
