# Supplementary figures and images for: Correction: The New Face of the Old Molecules: Crustin Pm4 and Transglutaminase Type I Serving as RNPs Down-Regulate Astakine-Mediated Hematopoiesis
Source: PLoS One. 2017 Jul 27;12(7):e0182405. doi: 10.1371/journal.pone.0182405 (PMC5531430; doi:10.1371/journal.pone.0182405)

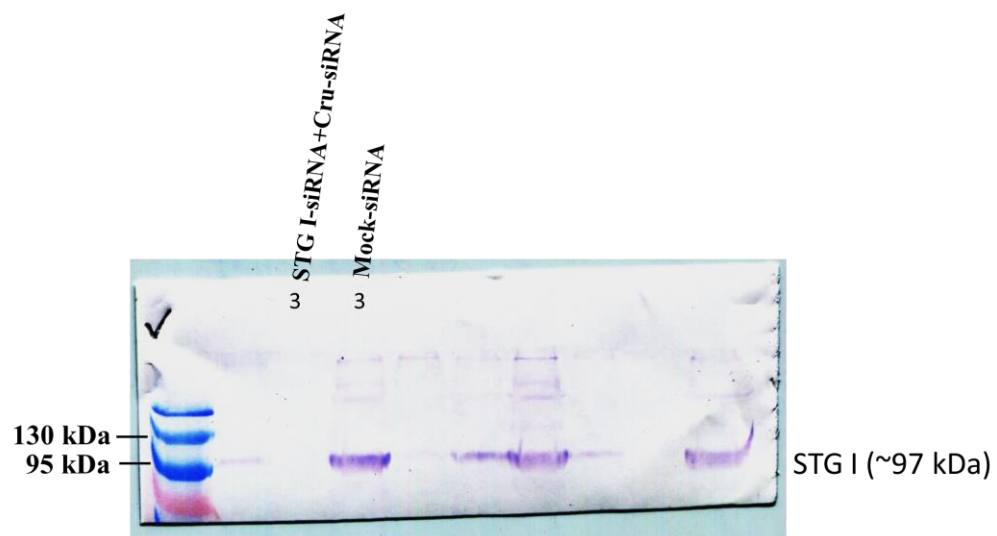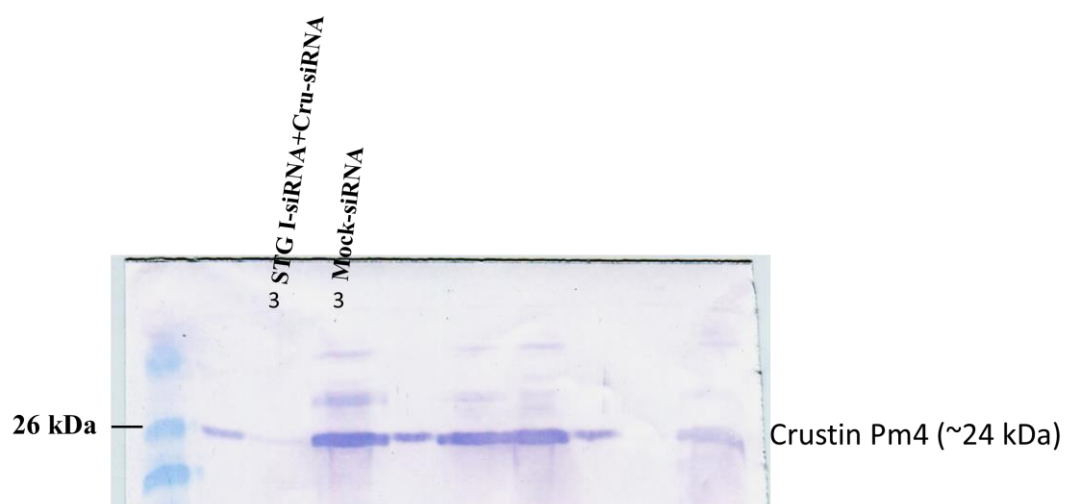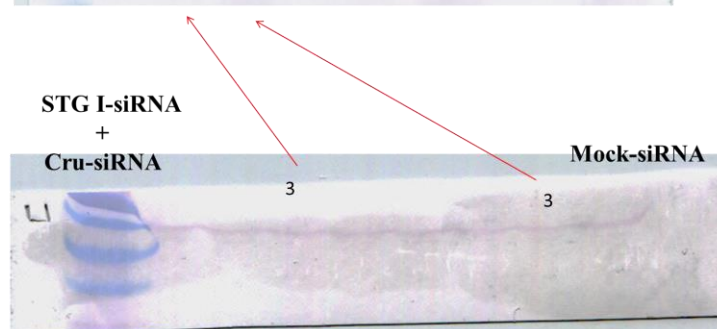

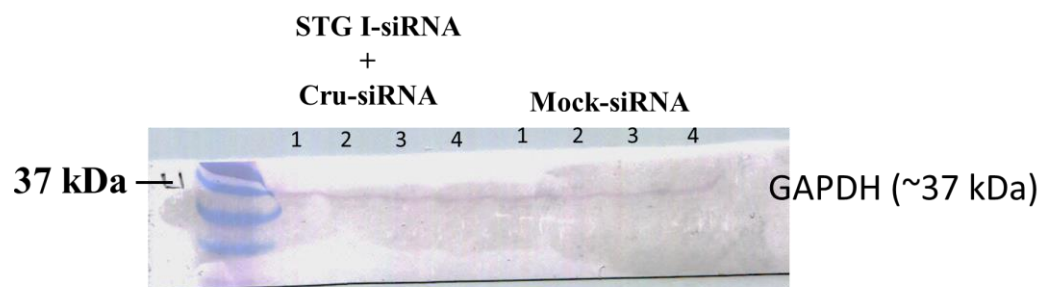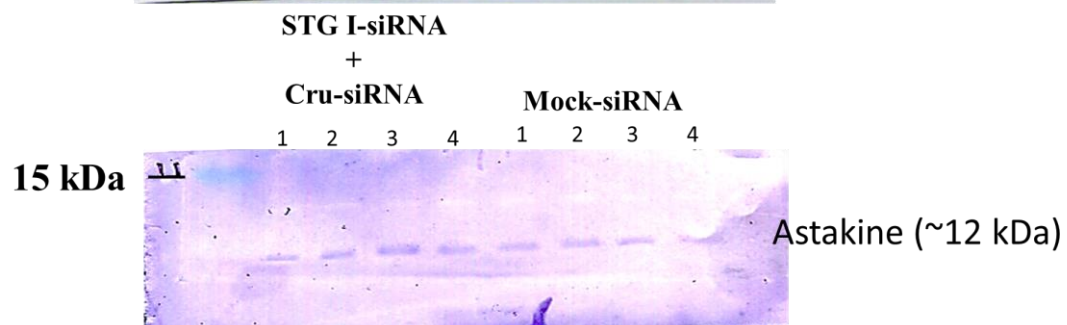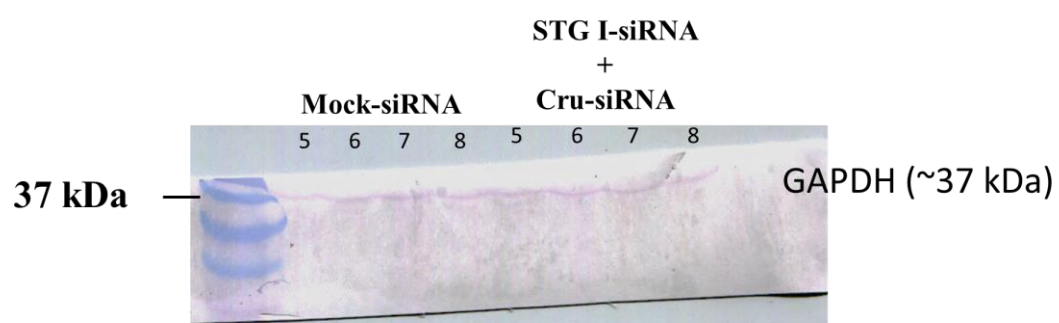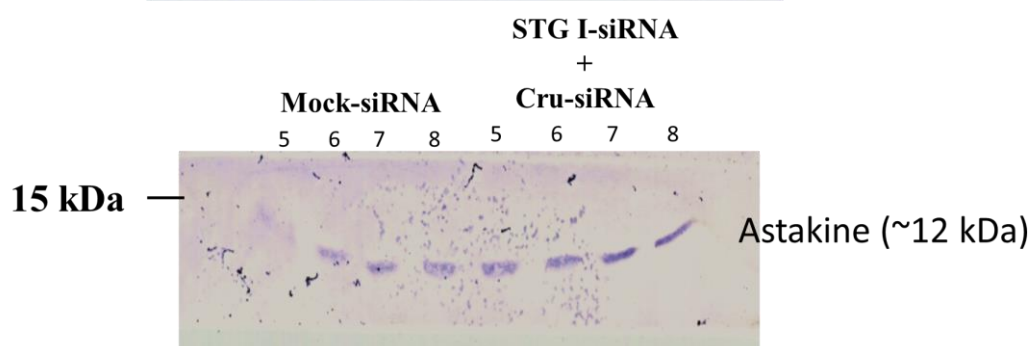

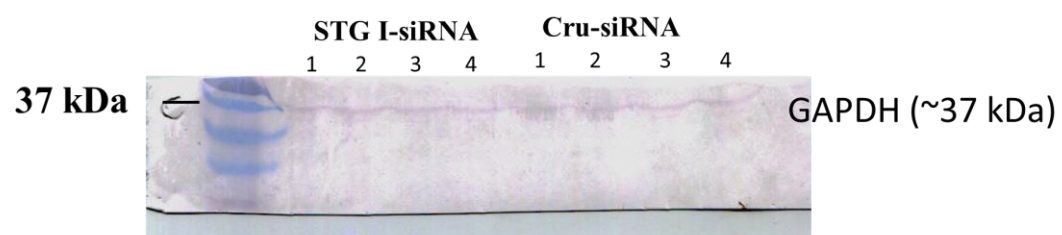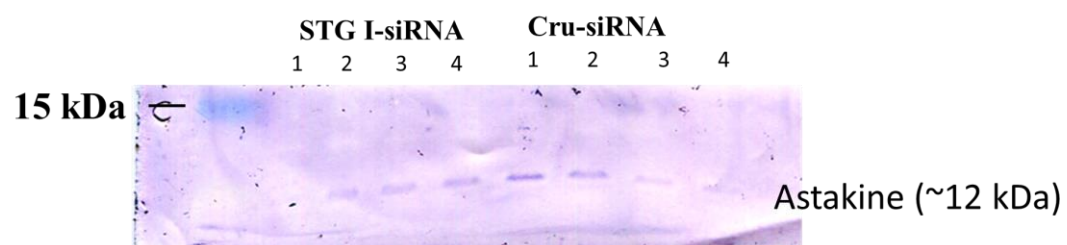

Supplement: S1 File — (PDF) [file pone.0182405.s001.pdf]
